# Intraspecific Variation in Drought and Nitrogen-Stress Responses in Pedunculate Oak (*Quercus robur* L.) Half-Sib Progeny

**DOI:** 10.3390/plants14243814

**Published:** 2025-12-15

**Authors:** Tatiana A. Grodetskaya, Anna A. Popova, Vladlena S. Ryzhkova, Ekaterina I. Trapeznikova, Petr M. Evlakov, Vadim G. Lebedev, Konstantin A. Shestibratov, Konstantin V. Krutovsky

**Affiliations:** 1 G.F. Morozov Voronezh State University of Forestry and Technologies, 394036 Voronezh, Russia; tatyana.pokusina@yandex.ru (T.A.G.); logachevaaa@rambler.ru (A.A.P.); vladlena.r11@yandex.ru (V.S.R.); katena.trapeznikova.02@mail.ru (E.I.T.); peter.evlakov@yandex.ru (P.M.E.); 2 Branch of the Shemyakin and Ovchinnikov Institute of Bioorganic Chemistry of the Russian Academy of Sciences, 142290 Pushchino, Russia; vglebedev@mail.ru (V.G.L.); schestibratov.k@yandex.ru (K.A.S.); 3 Department of Forest Genetics and Forest Tree Breeding, Georg-August University of Göttingen, 37077 Göttingen, Germany; 4 Laboratory of Population Genetics, N. I. Vavilov Institute of General Genetics, Russian Academy of Sciences, 119333 Moscow, Russia; 5 Laboratory of Forest Genomics, Genome Research and Education Center, Department of Genomics and Bioinformatics, Institute of Fundamental Biology and Biotechnology, Siberian Federal University, 660041 Krasnoyarsk, Russia

**Keywords:** CAT, carotenoids, chlorophyll, half-sibs, morphometry, nitrogen limitation, oak, POD, proline, *Quercus*, relative water content, ROS, SOD, water stress

## Abstract

Pedunculate oak (*Quercus robur* L.) face increasing threats from drought and nutrient limitation under climate change, yet their genetic variation may have adaptive potential. We examined the responses of pedunculate oak (*Quercus robur* L.) half-sib progeny from five maternal trees (1, 12, 32, 57, and 60) to water stress (WS), nitrogen limitation (NL), and combined WS+NL. WS reduced leaf relative water content (RWC) by 18–32% in all families and decreased proline only in two families (233.57 and 209.1), while four families (63.12, 149.1, 303.32, and 339.57) showed 1.7–2.0-fold proline accumulation. Exposure to WS+NL inhibited height and diameter growth in family 339.57 and diameter growth in families 23.12, 303.32 and 405.60, relative to the control. NL decreased chlorophyll (Chl) in two families (23.12 and 405.60) 1.5-fold and increased carotenoids in one of them (405.60) and RWC by 29% and 12% in 23.12 and 303.32 families, respectively. ROS-scavenging activities of catalase (CAT), guaiacol-dependent peroxidase (POD), and superoxide dismutase (SOD) enzymes increased 1.4- to 26.7-fold across all families except 151.1. Overall, families 303.32 and 339.57 were the most resilient to WS, NL, and WS+NL, whereas 233.57 and 151.1 were the most sensitive to WS, 23.12 to NL, and 405.60 to both stresses. These results highlight the family-level variation in stress responses and provide a basis for selecting resilient oak genotypes for forestry and conservation.

## 1. Introduction

Abiotic stresses are major factors of forest productivity and survival. Among them, drought and nitrogen (N) limitation (NL) are increasingly recognized as co-occurring and interactive global challenges, constraining growth by lowering photosynthetic capacity and resilience [1]. Climate change projections predict more frequent and intense drought episodes across temperate and Mediterranean regions [2,3], while global N cycling is simultaneously altered by anthropogenic deposition and chronic soil depletion [4,5]. Together, these factors shape photosynthesis, water relations, nutrient allocation, and oxidative stress responses in plants.

Oaks (*Quercus* spp.) represent cornerstone species of many ecosystems, from temperate deciduous forests to Mediterranean woodlands. They play pivotal roles in biodiversity support, timber production, and carbon sequestration [6]. However, oaks are vulnerable to combined water and nutrient stresses. Studies have shown that drought induces pigment degradation, proline accumulation, and antioxidant enzyme activation and reduces relative water content (RWC) [7,8]. Morphological growth traits integrate physiological adjustments over time. Oaks under drought and NL exhibit reduced biomass, leaf area, and acorn mass [3,9]. Root:shoot ratios may increase under moderate drought, but under combined drought and NL, both shoot and root biomass can collapse [10].

Chlorophyll (Chl) content declines consistently under drought and NL. In *Q. robur* provenances, drought caused substantial Chl *a* and *b* loss, particularly in mesic-origin populations [3]. In *Q. mongolica* seedlings, NL reduced Chl biosynthesis, restricted growth, and triggered the supply of N released during leaf senescence to maintain the photosynthetic apparatus [5]. Moreover, NL indirectly exacerbated water stress (WS) by impairing root development and osmotic adjustment [5]. Carotenoids, in contrast, are often preserved or relatively increased, raising the carotenoid-to-Chl ratio, which reflects enhanced photoprotection [7]. Moderate N supplementation can mitigate Chl decline, but severe NL accelerates pigment degradation [5].

RWC and pigment content are considered key indicators in determining drought tolerance in woody plants [10]. Drought reduced RWC in *Q. robur*, with provenances from mesic climates suffering sharper declines than those from drier native ranges [7]. In four Chinese oak species, *Quercus fabri* Hance, *Q. serrata* Thunb., *Q. acutissima* Carruth., and *Q. variabilis* Bl., RWC decreased under continuous drought stress, and showed an upward trend after rehydration [7]. Combined drought and NL led to the strongest declines in RWC and survival in *Q. variabilis* [11].

Drought stress consistently induces proline accumulation in oaks [7,8]. Comparative studies reveal that drought-adapted provenances accumulate proline earlier and to higher levels than mesic provenances [7]. Recent studies also suggest N status modulates proline dynamics. In *Q. dentata*, N enrichment under drought supported higher proline accumulation compared with N-deficient plants, improving stress tolerance [9]. NL constrains maximal proline synthesis because of its nitrogenous nature, with severe N starvation preventing sufficient proline accumulation [5]. This highlights the interaction between nutrient status and osmolyte synthesis pathways.

Reactive oxygen species (ROS) accumulate under both drought and N starvation. Oaks respond with increased activity of ROS-scavenging enzymes. Drought-induced increases in catalase (CAT), guaiacol-dependent peroxidase (POD), and superoxide dismutase (SOD) have been reported in multiple oak species [7,8]. SOD activity generally increased under drought in *Q. robur*, with greater induction in drought-adapted provenances [7,9].

POD is strongly upregulated under drought, while dry-site provenances often show higher basal and inducible POD activity [8]. CAT activity was shown to be more variable, with some provenances showing transient increases and others declines under prolonged drought [7]. However, NL alters this response, while severe NL reduces antioxidant enzyme capacity [5]. Field data on Mediterranean oaks showed that lower foliar nutrient status was associated with reduced Chl and altered oxidative-stress indicators (H_2_O_2_, lipid peroxidation) and changes in antioxidant enzymes such as SOD, CAT, and POD [12]. Similarly, *Q. dentata* seedlings under drought showed stronger antioxidant responses when N was sufficient [9]. This indicates that N availability shapes the oxidative stress buffering capacity of oaks.

Half-sib lines of *Q. robur* differ significantly in biometrical, anatomical, and physiological traits under drought, including root:shoot ratio, leaf thickness, stomatal density, and photochemical efficiency [13]. However, how WS and NL interactively affect physiological variation at the half-sib family level in oak and whether the stress-resistant traits of plus trees are stably inherited in their progeny remains unclear.

Population-level studies reveal geographic variation in drought tolerance [14] and high heritability of phenological traits in northern oak populations [15]. Drought timing further modulates growth and leaf development in seedlings [16]. At the same time, genomic studies link leaf phenology with adaptive drivers such as heat stress and biotic pressures [17]. Pre-acclimation experiments suggest that moderate prior stress can enhance tolerance to subsequent drought, as shown for *Q. ilex* [18].

For forest management, provenance selection emerges as a critical strategy: dry-site provenances demonstrate smaller RWC declines, higher proline accumulation, stronger antioxidant responses, and more resilient pigment profiles. Moderate N supplementation can buffer drought impacts, though ecological trade-offs such as altered competition and leaching must be considered [19].

Although drought and NL are major stress factors for forestry and oak ecosystems on a global scale, studies analyzing the physiological and biochemical responses of the genus *Quercus* under NL, particularly in combination with water deficit, remain scarce. Existing studies do not adequately address intraspecific variation or trace the transmission of stress-resistance traits from plus trees to their progeny. In this study, we analyzed height and diameter growth, as well as physiological and biochemical responses—including changes in RWC, proline accumulation, pigment composition, and the activity of ROS-scavenging enzymes (CAT, POD, and SOD)—under controlled partial stresses of WS and NL, both separately and in combination, in F_2_ progeny of plus trees of *Q. robur*. Our findings highlight the necessity of incorporating physiological and biochemical evaluations into plus-tree selection programs.

## 2. Results

### 2.1. Leaf Pigment Content

**Figure 1:**
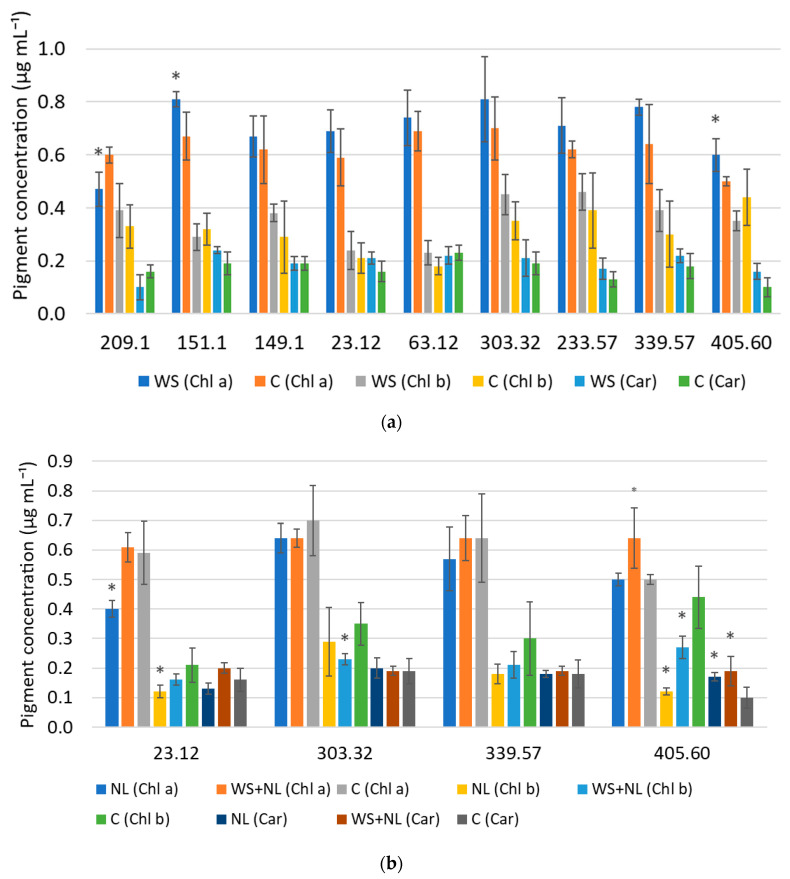
Leaf pigment concentrations in oak families under (**a**) water stress (WS), (**b**) nitrogen limitation (NL) and combined stress (WS+NL). Control (C) represents well-watered plants supplied with sufficient nitrogen. Chl *a*—chlorophyll *a*, Chl *b*—chlorophyll *b*, Car—total carotenoids; * indicates statistically significant differences in pigment concentration of WS-, NL- or WS+NL-treated oak plants relative to the control (*p* < 0.05 according to Fisher’s LSD test). Five plants from each family were sampled for each treatment and control. Bars represent standard deviation (±SD).

WS had no significant effect on total chlorophyll content overall, but induced family-specific changes in the chlorophyll *a*:*b* and carotenoid ratios. NL reduced pigment concentrations in some families, while combined stress (WS+NL) produced mixed responses. WS did not affect the total Chl content; however, it increased Chl *a* concentration in families 151.1 and 405.60 by 17% and 20%, respectively (Figure 1a). At the same time, the Chl *a*:*b* ratio in family 405.60 increased 1.5-fold compared to the control. In contrast, Chl *a* content in family 209.1 decreased by 22% under WS. The levels of Chl *b* and carotenoids (Car) in these families remained unchanged compared to the control. However, the Car:Chl ratio increased 1.6- and 1.5-fold in families 405.60 and 209.1, respectively. In families 63.12, 233.57, 23.12, 303.32, and 339.57, increases in Chl *a* were observed, but they were not statistically significant.

NL reduced the concentrations of total Chl in 23.32 and 405.60, but not in 303.32 and 405.60 (Figure 1b). In family 23.12, Chl *a* and Chl *b* contents decreased 1.5- and 1.8-fold under NL, respectively, while Car remained unchanged. In family 303.32, Chl *b* decreased 1.5-fold under WS+NL. In family 405.60, Chl *a* increased by 28% under WS+NL, whereas Chl *b* declined 3.7-fold under NL and 1.6-fold under WS+NL. Car content in this family increased 1.7-fold under NL and 1.9-fold under WS+NL. In family 339.57, decreases in Chl *a* and *b* under NL and WS+NL were not statistically significant, and Car levels remained at the control level. Under NL, the Chl *a*:*b* ratio increased 1.5- and 3.7-fold in families 339.57 and 405.60, respectively, whereas under WS+NL it increased 1.4-fold in families 23.12, 303.32, and 339.57, and 2.1-fold in family 405.60. Neither NL nor WS+NL significantly affected the Car:Chl ratio in families 23.12, 303.32, and 339.57, while in family 405.60 it increased 2.6- and 2.0-fold, respectively.

### 2.2. Relative Water Content (RWC)

**Figure 2:**
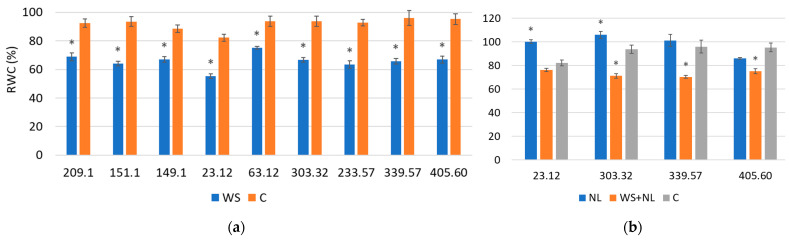
Relative water content (RWC) in oak leaves after exposure to (**a**) water stress (WS), (**b**) nitrogen limitation (NL) and combined stress (WS+NL). Control (C) represents well-watered plants with sufficient nitrogen; * indicates statistically significant differences in RWC in WS-, NL- or WS+NL-treated oak plants relative to the control (*p* < 0.05 according to Fisher’s LSD test). Five plants from each family were sampled for each treatment and control. Bars represent standard deviation (±SD).

WS significantly reduced RWC in all families, but the magnitude of decline varied, with some families showing stronger resistance. NL did not reduce RWC and in certain cases even increased it, while combined WS+NL generally mitigated the reductions seen under WS alone. Exposure to WS significantly reduced leaf RWC in all oak families examined. Families 63.12 and 23.12 were the least affected, with RWC decreasing by 18% and 23%, respectively (Figure 2a). By contrast, the most sensitive families (405.60, 233.57, 339.57, and 151.1) showed declines by 30–32%, reaching the lowest values among the families studied.

NL did not reduce RWC in any of the studied families (Figure 2b). On the contrary, a 29% increase was observed in family 23.12 and a 12% increase in family 303.32. No statistically significant differences from the control were observed in families 339.57 and 405.60. Under WS+NL, reductions in RWC were less pronounced than under WS alone. For example, in families 303.32 and 339.57, RWC was 5% higher under WS+NL compared with WS alone. In family 23.12, no difference in RWC was found between WS+NL and the control.

### 2.3. Proline Content

**Figure 3:**
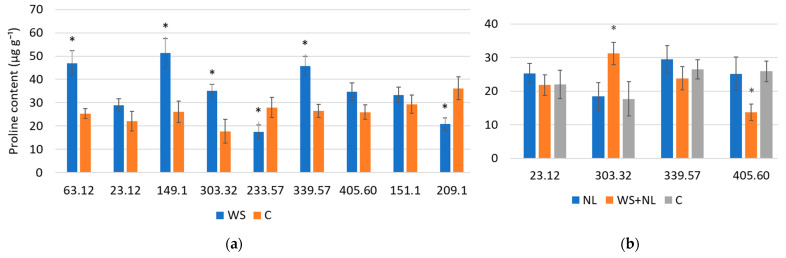
Proline content in oak leaves under (**a**) water stress (WS), (**b**) nitrogen limitation (NL) and combined stress (WS+NL). Control (C) represents well-watered plants with sufficient nitrogen; * indicates statistically significant differences in proline content in WS-, NL- or WS+NL-treated oak plants relative to the control (*p* < 0.05 according to Fisher’s LSD test). Five plants from each family were sampled for each treatment and control. Bars represent standard deviation (±SD).

Proline accumulation was induced by WS in several families, but decreased in others, highlighting contrasting metabolic responses. NL alone had little effect, while WS+NL produced family-specific changes. Relative proline content increased by 1.7–2.0-fold under WS in families 63.12, 149.1, 303.32, and 339.57 (Figure 3a). In families 23.12, 405.60, and 151.1, the increase was not statistically significant, while in families 233.57 and 209.1 proline decreased 1.6- and 1.7-fold, respectively.

NL did not significantly alter proline levels in any family, which remained at control values (Figure 3b). Combined WS+NL also did not affect proline in families 23.12 and 339.57. In family 405.60, however, proline decreased 1.9-fold relative to the control, while in family 303.32 it increased 1.8-fold under WS+NL, a response similar to WS alone.

### 2.4. ROS-Scavenging Enzyme Activity

**Figure 4:**
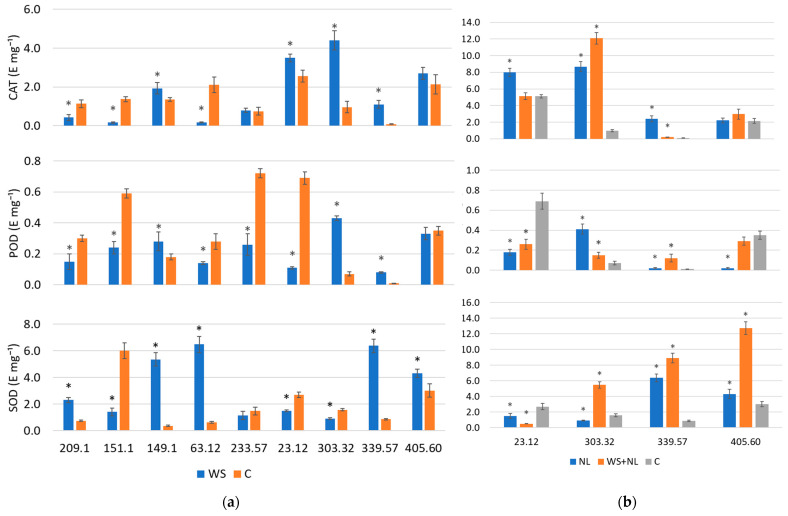
Activity of ROS-scavenging enzymes in oak leaves under (**a**) water stress (WS), (**b**) nitrogen limitation (NL) and combined stress (WS+NL). Control (C) represents well-watered plants with sufficient nitrogen; * indicates statistically significant differences in ROS-scavenging enzymes activity in WS-, NL- or WS+NL-treated oak plants relative to the control (*p* < 0.05 according to Fisher’s LSD test). Five plants from each family were sampled for each treatment and control. Bars represent standard deviation (±SD).

Antioxidant enzyme activities (CAT, POD, SOD) showed strong family-specific variation. Some families exhibited large-fold increases under WS, NL, or WS+NL, while others showed little or no activation. Responses of antioxidant enzymes to WS varied among families. In family 149.1, CAT, POD, and SOD activities increased 1.4-, 1.6-, and 14.9-fold, respectively. In family 339.57, the respective increases were 12.2-, 8.0-, and 7.5-fold (Figure 4a).

In family 339.57, NL increased CAT, POD, and SOD activities 26.7-, 2.0-, and 7.5-fold, respectively. Combined WS+NL further increased their activities 2.2-, 12.0-, and 10.5-fold, respectively (Figure 4b). In family 303.32, CAT and POD activities increased 4.6- and 6.1-fold under WS, respectively. These enzyme activities rose 9.0- and 5.9-fold under NL, and 12.6- and 2.1-fold under WS+NL. SOD activity in this family increased only by 3.5-fold under WS+NL. SOD activity also increased under WS in families 209.1 and 63.12 3.2- and 10.5-fold, respectively. In family 405.60, SOD activity increased 1.4-fold under WS, 1.4-fold under NL, and 4.2-fold under WS+NL. In family 23.12, CAT activity increased 1.4-fold under WS and 1.6-fold under NL. In contrast, families 151.1 and 233.57 showed no significant increases in CAT, POD, or SOD activity under WS treatment.

### 2.5. Plant Height and Diameter

**Figure 5:**
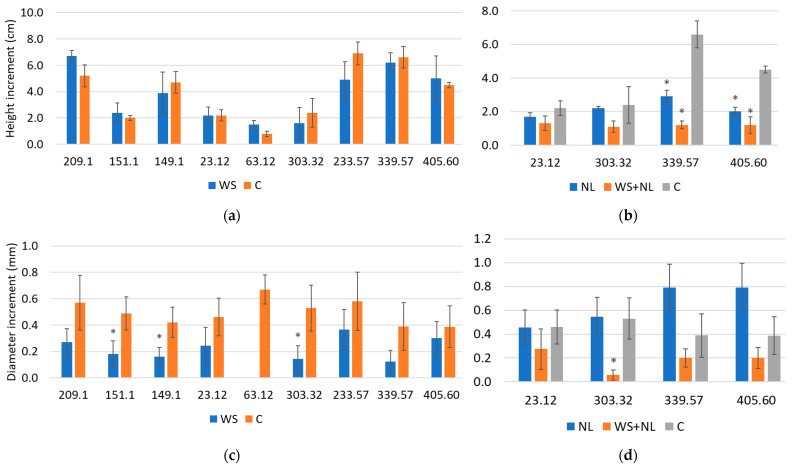
Height (**a**,**b**) and diameter (**c**,**d**) increments of oak seedlings under water stress (WS), nitrogen limitation (NL), and combined WS+NL. Control (C) represents well-watered plants supplied with sufficient nitrogen. Height and diameter were measured before (T1) and after (T2) treatments, and increment was calculated as T2–T1. Values represent mean ± SD; * indicates statistically significant differences based on five seedlings per family in each treatment and control (*p* < 0.05, Fisher’s LSD test).

WS negatively affected diameter increment (Figure 5c) but did not significantly alter height increment (Figure 5a) across the analyzed oak families. In contrast, NL did not reduce diameter increment (Figure 5d) but decreased height increment in certain families (Figure 5b). Combined WS+NL stress also led to pronounced reductions in increment traits in several families (Figure 5b,d).

Specifically, families 151.1, 149.1, and 303.32 showed 2.6-, 2.7-, and 3.7-fold reductions in diameter increment relative to the control, respectively (Figure 5c). In family 63.32, no diameter increment was observed under WS (Figure 5c), whereas control plants of this family increased by 0.7 ± 0.11 mm.

NL treatment significantly reduced height increment in families 339.57 and 405.60, by 2.3-fold compared with plants supplied with sufficient N (Figure 5b). The diameter increment in these families under NL was not statistically significant. In families 23.12 and 303.32, NL had no detectable effect on either height or diameter increment (Figure 5b,d).

Combined WS+NL stress caused statistically significant reductions in height increment in families 339.57 and 405.60, by 5.5- and 3.8-fold, respectively (Figure 5b). A strong reduction in diameter increment was also observed in family 303.32, with a 9.2-fold decrease compared with the control (Figure 5d).

Overall, families 233.57, 405.60, and 151.1 were the most sensitive to WS, showing strong reductions in RWC, limited proline accumulation, and weak antioxidant activation. Family 23.12 was most sensitive to NL, with marked pigment declines. By contrast, families 149.1 and 63.12 were WS-resistant, while 303.32 and 339.57 displayed the broadest adaptive responses to both stresses, maintaining pigment content and activating antioxidant defenses.

## 3. Discussion

The present study confirmed that WS significantly reduced leaf tissue hydration and height growth in all analyzed families, consistent with previous reports that drought reduces photosynthesis, stem diameter increment, and height growth. Under moderate drought, however, the negative impact of suboptimal N on growth traits such as height increment was less pronounced, although acorn mass was strongly affected by low N in both wet and dry years [3]. These findings suggest that drought (WS) is generally a stronger stressor than NL for growth traits, although adequate N availability remains essential for growth and reproductive performance.

We acknowledge that in some families (e.g., 233.57) proline levels decreased under WS. This may reflect family-specific differences in N metabolism and stress adaptation. Proline accumulation typically depends on active N assimilation and diversion of carbon and N into amino acid biosynthesis. Therefore, reduced proline could indicate impaired N uptake or assimilation under stress [20]. Alternatively, decreased proline may result from enhanced utilization of proline as an energy and redox buffer during stress responses [21,22]. Similar variability in proline dynamics under abiotic stress has been reported in crop species [23]. Thus, the observed decline likely reflects variation in metabolic regulation among families rather than a uniform response.

In family 23.12, the increase in RWC under NL likely reflects reduced transpiration demand (via stomatal downregulation and smaller leaf area) together with osmotic adjustment (accumulation of compatible solutes and K^+^), which enhances turgor maintenance and water retention [20,21]. Structural changes (e.g., increased leaf thickness and altered cell wall elasticity) may further contribute to higher tissue water content per unit dry mass. Similar responses have been described under nutrient limitation and osmotic stress, where stomatal regulation and osmotic adjustment improve instantaneous water status despite growth constraints [20,24]. Thus, NL can improve instantaneous water status even as it constrains growth, providing a plausible explanation for the observed RWC increase.

Growth responses under NL varied among half-sib families, with some showing diameter increment while others did not. This pattern may be explained by anatomical plasticity, reflecting family-specific differences in cambial activity and the rate of wood tissue formation under stress. Such variation in radial growth traits can serve as diagnostic indicators of stress resilience. For example, in Norway spruce, latewood proportion (LWP) has been successfully applied to discriminate drought-tolerant half-sib families [25]. Recent studies further highlight the role of anatomical and physiological plasticity in stress adaptation: maize root and shoot systems exhibit plastic responses under NL that optimize resource use [26,27], while integrated morphological and physiological plasticity has been reported in conifers under varying NH_4_^+^:NO_3_^−^ ratios [28]. In trees, cambial phenology and xylogenesis are highly sensitive to environmental stress, with divergence in cambial activity linked to hydraulic adjustment and wood anatomical changes [29,30]. Taken together, these findings support the interpretation that diameter increment under NL in family 23.12 may reflect adaptive anatomical plasticity and differential cambial activity, enabling certain families to maintain radial growth despite reduced nutrient availability.

The family-level variation observed in our material is consistent with reported heritability values in oaks, which are generally moderate (*h*^2^ ≈ 0.2–0.4 for growth and phenology). Such values imply that meaningful genetic gain (10–15%) can be achieved through selection [15]. Genomic approaches further demonstrate the potential to accelerate gain in wood quality traits such as heartwood formation [31]. Therefore, the variation that we reported here likely reflects heritable differences in stress adaptation, supporting the potential use of these traits in oak breeding programs.

Importantly, tolerance to WS does not imply tolerance to NL, as these responses are partly decoupled [20]. WS responses are driven by hydraulic regulation, stomatal control, xylem architecture, and osmotic adjustment, whereas NL alters nitrate transport, assimilation, and carbon–nitrogen partitioning. This decoupling explains why some families maintained performance under WS but not under NL, underscoring the need to evaluate these stress responses independently.

The observed changes in CAT, SOD, and POD activities should be viewed within the broader antioxidant network. Non-enzymatic antioxidants, including ascorbate and glutathione (central to the ascorbate–glutathione cycle), flavonoids, and tocopherols, act in concert with enzymes to scavenge reactive oxygen species and regenerate redox balance [32,33,34]. Although not measured here, family-specific variation in these metabolites may modulate the efficiency of enzymatic defenses and contribute to differences in stress responses.

Future studies should employ transcriptomic and metabolomic approaches to resolve family-specific stress mechanisms. ABA biosynthesis and signaling are pivotal for stomatal control and drought acclimation, while the ascorbate–glutathione cycle underpins redox buffering and ROS detoxification [23,35,36,37]. Recent analyses of ascorbate metabolism and pathway control provide tractable gene sets for targeted profiling (e.g., GDP-L-galactose phosphorylase, dehydroascorbate reductase, key ABA biosynthetic and signaling regulators).

## 4. Conclusions

The presented study revealed pronounced variation among *Q. robur* half-sib F_2_ families in their physiological and biochemical responses to WS, NL, and combined WS+NL. WS had a stronger inhibitory effect on growth and pigment accumulation than NL, consistent with previous findings that water limitation is a major constraint for oak establishment under climate change scenarios. Families 303.32 and 339.57 exhibited higher RWC, increased proline accumulation, and stronger activation of ROS-scavenging enzymes (CAT, POD, and SOD) under WS, suggesting efficient osmotic adjustment and oxidative stress regulation. In contrast, families 405.60, 233.57, and 151.1 showed reduced hydration and pigment degradation, indicating lower WS tolerance. NL alone induced moderate declines in chlorophyll content and growth, but when combined with WS, it intensified stress symptoms, confirming the interactive nature of abiotic stress factors.

From a practical perspective, half-sib families maintaining higher RWC and antioxidant capacity under WS are promising candidates for reforestation and nursery programs in drought-prone areas. Breeding selection should prioritize resilient families such as 303.32, 339.57, and 63.12, which exhibited consistent stability across treatments. Further research integrating genetic, transcriptomic, and metabolomic analyses is needed to elucidate the molecular mechanisms underlying the observed physiological variability and to assess long-term performance under field conditions. The coordinated activation of antioxidant enzymes together with proline accumulation in resilient families may reflect more efficient stress signaling and resource allocation strategies, enabling simultaneous osmotic adjustment and ROS detoxification. Overall, the results highlight that intra-specific diversity among oak half-sibs represents a valuable resource for developing drought- and nutrient-efficient genotypes suitable for sustainable forestry under changing climate conditions.

## 5. Materials and Methods

### 5.1. Half-Sibs F_2_ Progeny Origin and Plant Material

The objects of the study were two-year-old seedlings representing open-pollinated half-sib progeny of the second generation (F_2_) from plus trees of pedunculate oak (*Q. robur*). The acorns were collected in autumn 2022 from a seed orchard of half-sib F_1_ progeny of plus trees established by Yu. P. Efimov in 1976 and located on the territory of the Semiluki Forest Breeding Experimental and Demonstration Nursery in the Voronezh region, Russia (Table 1). This plus tree seed orchard was established by sowing acorns harvested under the crowns of late-blooming and intermediate-blooming plus trees growing in the Shipov Forest located on the right bank of the Osered River, Voronezh Region near the watershed with the Bityug River (coordinates: 50°46′00″ N, 40°20′00″ E). Among these trees representing F_1_ progeny of plus trees from the Shipov Forest, trees with the highest productivity assessed by trunk diameter, good vitality, and high seed production were selected for the F_2_ progeny experiment.

The F_2_ half-sib families were confirmed on the basis of maternal identity. The origin of the maternal plus trees is the Krasnoye forestry of the Vorontsov Forest Enterprise, Voronezh region; the forest site conditions are type D2 (fresh oak woodland).

### 5.2. Climatic Context

According to the worldwide bioclimatic classification system [38], the Semiluki Forest Breeding Station (Voronezh region, Russia) falls within the temperate continental bioclimate, supratemperate thermotype. Based on the long-term observations at the Voronezh Meteorological Station average temperatures for the period of 1861–2024 are +19.2 °C for summer, −7.4 °C for winter, and +6.0 °C annual. Average temperatures for 2024 are +22.4 °C for summer, −4.2 °C for winter, and +9.2 °C annual. Average annual precipitation is 575 mm. The regional climate is characterized by warm, partly cloudy summers and long, cold winters that are snowy, windy, and overcast. Over the course of the year, temperatures typically range from −11 °C to +26 °C, rarely falling below −23 °C or exceeding +33 °C [39].

### 5.3. Edaphic Context

Maternal trees of *Q. robur* originated from typical chernozem soils (according to FAO/WRB classification—haplic chernozem). These soils are rich in organic matter, with humus contents of 6–9% (light mechanical composition 4–5%; 3–6% in marginal areas). Humus decreases gradually with depth, and humic acids dominate over fulvic acids (C_h_:C𝒻 ≈ 2). Soil reaction is neutral (pH 7.0–7.5), with high cation exchange capacity (35–55 meq per 100 g soil). Calcium strongly predominates among exchangeable bases, while magnesium is present in lower proportions. The overall mineral composition is uniform, and clay content is evenly distributed throughout the profile.

### 5.4. Controlled Experimental Conditions

The acorns were sown into 4 L vegetation containers filled with a growth medium consisting of sphagnum peat neutralized with lime and dolomite flour, mixed with perlite in a 3:1 ratio. The substrate was adjusted to a pH of 5.5–6.5 and an electrical conductivity of 0.0–0.5 dS·m^−1^. In addition, the medium contained Fertica Plus, providing macronutrients with N 2.5–4% (including 1.5–2.5% in available form), phosphorus (P) 0.3–0.4%, and potassium (K) 0.5–0.6%. Chelated micronutrients were also present, including boron (B) 0.02%, copper (Cu) 0.01%, iron (Fe) 0.1%, manganese (Mn) 0.1%, and zinc (Zn) 0.01%.

Seedlings were grown in greenhouse conditions with controlled temperature, humidity, and photoperiod. Daytime temperatures were maintained at 22–25 °C, nighttime at 18–20 °C, with relative humidity of 60–70%. Photoperiod was set to 16 h light/8 h dark, using supplemental lighting to ensure uniform irradiance. These conditions were chosen to minimize environmental variability and isolate the effects of WS, NL, and combined WS+NL treatments.

### 5.5. Measurements

The height and root collar diameter of each oak seedling were measured immediately before the WS and NL treatments and again at the end of the experiment. Height was recorded with a ruler with 0.1 cm precision, and diameter was determined with a digital caliper with 0.1 mm precision. Values are presented as means per each family for each measurement.

### 5.6. Determination of Soil Water-Holding Capacity

To maintain the required values of the soil moisture, full soil water-holding capacity (FWHC) was determined before the start of the experiment [40]. The soil was sifted and poured into a cylinder 3–4 cm in diameter and 10–20 cm high (according to the height of the growing vessel), the lower end was tied with gauze holding the filter paper. At the same time, a soil sample was taken to determine the moisture content (drying at 105 °C). The soil was poured, compacting it with light tapping, to a level of 1–2 cm below the upper edge. The cylinder was placed in water (the level was 5–7 mm above the lower soil level), covered to prevent evaporation and kept until completely saturated. Then, it was taken out of the water, dried on the outside and placed on filter paper. As soon as the water stopped draining, the cylinder was weighed on an analytical balance and placed in a crystallizer under a hood for 1–2 h and weighed again. This operation was repeated until the weight of the cylinder with soil that had absorbed the water became constant. FWHC was calculated as the ratio of the difference between the mass of saturated and dry soil to the mass of dry soil, expressed as a percentage.

### 5.7. Water Stress (WS) and Nitrogen Limitation (NL) Treatments

For the experimental setup, plants were divided into four groups. The first group was subjected to WS (water deficit), the second to NL, and the third to a combined WS and NL (WS+NL), while the fourth group served as the control under optimal growth conditions.

WS was simulated by growing oak seedlings under reduced soil moisture. In the control treatment, soil moisture was maintained at 80 ± 5% of full water-holding capacity (FWHC), while in the WS treatment it was adjusted to 45 ± 5%. WS treatment was maintained for six weeks. Prior to stress imposition, all plants were watered uniformly to maintain 75–85% FWHC. Pots were weighed three times per week, and evaporative water loss was replenished individually for each pot. At the onset of WS treatment, watering was withheld until soil moisture reached 40–45% FWHC. Thereafter, WS plants were maintained at 40–50% FWHC, while control plants remained at 75–85% FWHC.

Following the approach used in [41] plants were supplied with two variants of Hoagland’s nutrient solution, for the N-sufficient (N^+^) and N-limitation (N^−^) treatments. Fertilization was applied weekly for six weeks prior to WS and continued throughout the WS period. For the N-sufficient treatment (N^+^), the final concentrations of macronutrients were: KNO_3_ (6 mM), Ca(NO_3_)_2_·4H_2_O (4 mM), MgSO_4_·7H_2_O (1 mM), and NH_4_H_2_PO_4_ (1 mM). Micronutrients were added as H_3_BO_3_ (46 µM), MnCl_2_·4H_2_O (9 µM), ZnSO_4_·7H_2_O (0.8 µM), CuSO_4_·5H_2_O (0.3 µM), and Na_2_MoO_4_·2H_2_O (0.25 µM). N was supplied as a mixture of nitrate (NO_3_^−^) and ammonium (NH_4_^+^), with a NO_3_^−^:NH_4_^+^ ratio of approximately 10:1.

For the nitrogen-limitation treatment (N^−^), macronutrients were provided as K_2_SO_4_ (3 mM), CaSO_4_ (2 mM), MgSO_4_·7H_2_O (2 mM), and Ca(H_2_PO_4_)_2_ (0.5 mM). Micronutrients were added at the same concentrations as in the N^+^ solution. Both N^+^ and N^−^ solutions contained Fe–Na–EDTA (100 µM). Stock solutions (macronutrients, micronutrients, and iron) were stored at 4 C and mixed immediately before use.

### 5.8. Enzyme Activity Assays

For enzyme analysis, 1 g of fresh leaf tissue was homogenized in 3 mL of sodium phosphate buffer (pH 7.8) containing 1 mM EDTA and 2% (*w*/*v*) polyvinylpolypyrrolidone (PVPP). The homogenate was centrifuged at 15,000× *g* for 40 min at 4 °C, and the resulting supernatant was used for protein quantification and enzyme activity assays [42].

CAT activity was determined by measuring the change in absorbance at 240 nm, according to [43]. The reaction was initiated by adding 200 μL of extract to 2.8 mL of a mixture containing 50 mM phosphate buffer (pH 7.0) and 15 mM H_2_O_2_. Absorbance was recorded every 30 s for 0.5–3 min. One unit of CAT activity was defined as the decomposition of 1 μmol H_2_O_2_ per min.

POD activity was assayed following the method of [44]. The reaction was initiated by adding 0.2 mL of extract to 2.8 mL of a mixture containing 0.25% guaiacol in 10 mM sodium phosphate buffer (pH 6.0) and 100 mM H_2_O_2_. Absorbance at 470 nm was measured every 30 s between 0.5 and 3 min. POD activity was expressed as μmol guaiacol oxidized per mg protein per min. Control samples contained buffer instead of supernatant. The rate of absorbance increase in the linear range was used for calculations.

SOD activity was determined based on the enzyme’s ability to inhibit the photochemical reduction in nitroblue tetrazolium (NBT), according to [45]. The reaction medium was prepared in phosphate buffer (pH 7.8) immediately before use. A fresh solution of riboflavin (4.4 mg per 100 mL distilled H_2_O) was prepared and stored in darkness at 4 °C.

Reaction mixtures were prepared in three types of tubes:

- dark control: 3 mL reaction medium + 0.2 mL supernatant;
- light control: 3 mL reaction medium + 0.2 mL phosphate buffer (pH 7.8), without supernatant;
- experimental tubes: 3 mL reaction medium + 0.2 mL supernatant.

The enzymatic reaction was initiated by adding 0.05 mL riboflavin to all tubes. The light control and experimental tubes were exposed to light for 15 min, while the dark control was kept at 30 °C without illumination. The reaction was terminated by transferring the tubes to darkness, and absorbance was recorded at 560 nm. SOD activity was expressed in relative units per mg protein.

Protein content was determined using the standard Lowry method [46]. Quantification was performed against a calibration curve prepared with bovine serum albumin (BSA) as the standard. Enzyme activities were reported as U mg^−1^ protein.

### 5.9. Pigment Content Determination

Chl *a* and *b* and carotenoid concentrations were determined following [47]. Approximately 100 mg of leaf tissue was ground in liquid N, mixed with 1 mL of 80% acetone, and incubated with shaking for 15 min in darkness. Samples were centrifuged for 5 min at 13,000 rpm, and the supernatant was transferred to light-protected tubes. The extraction was repeated twice, supernatants were pooled, and the final volume was adjusted to 3 mL. Absorbance was measured at 663, 646, and 470 nm against 80% acetone.

### 5.10. Relative Water Content (RWC) Assay

The RWC of oak leaves was determined according to the standard gravimetric method with slight modifications [48,49]. Fresh leaf disks (0.5–1.0 cm in diameter) were excised from fully expanded leaves and immediately weighed to obtain the fresh weight (FW). The disks were then floated on distilled water in Petri dishes and incubated under low light at room temperature for 16 h until full turgidity was achieved. After gently blotting the surface water with filter paper, the turgid weight (TW) was recorded. The samples were subsequently oven-dried at 70 °C for 48 h to obtain the dry weight (DW). RWC was calculated as the ratio of the difference between the fresh weight (FW) and the dry weight (DW) to the difference between the turgid weight (TW) and the dry weight (DW) and expressed as a percentage.

### 5.11. Proline Content Assay

Free proline content was determined according to the ninhydrin-based colorimetric method of Bates et al. [50] with slight modifications. Fresh leaf tissue (0.5 g) was homogenized in 10 mL of 3% (*w*/*v*) sulfosalicylic acid and centrifuged at 10,000× *g* for 10 min. Two milliliters of the supernatant were mixed with 2 mL of acid ninhydrin reagent and 2 mL of glacial acetic acid in a test tube. The reaction mixture was incubated in a boiling water bath at 100 °C for 1 h and then rapidly cooled on ice. The chromophore was extracted with 4 mL of toluene, and the absorbance of the organic phase was measured at 520 nm using a spectrophotometer. Proline concentration was calculated from a standard curve prepared with L-proline and expressed as µmol g^−1^ fresh weight.

### 5.12. Statistical Analysis

All data are presented as mean ± standard deviation (SD) of five biological replicates (n = 5), representing five independent seedlings per each family, 209.1, 151.1, 149.1, 23.12, 63.12, 303.32, 233.57, 339.57, and 405.60, in the control and water stress (WS) treatments. Five additional seedlings per family, 23.12, 303.32, 339.57, and 405.60, were also selected for an extended experiment including WS, NL, and combined WS+NL treatments. Statistical significance of differences between each treatment and the corresponding control within each oak family was evaluated using Fisher’s least significant difference (LSD) test at a probability level of *p* < 0.05. Standard deviations were calculated to assess variability within each treatment group. All statistical analyses were performed using Statistica v.10.0 (StatSoft Inc., Tulsa, OK, USA).

## Figures and Tables

**Table 1:** List of plus trees of pedunculate oak represented in the seed orchard established in 1976 at the Semiluki Forest Breeding Experimental and Demonstration Nursery (Voronezh region, Russia).

Tree No. (by Location)	Tree No. (State Register)	Location, Block	Height, m	Diameter, cm
1	19.1	57	34.5	66.5
12	25.12	57	34.5	52.0
32	–	35	34.5	50.0
57	165.60	33	33.5	60.0

## Data Availability

The original contributions presented in this study are included in the article. Further inquiries can be directed to the corresponding author.
